# Continuous positive airway pressure and noninvasive ventilation in prehospital treatment of patients with acute respiratory failure: a systematic review of controlled studies

**DOI:** 10.1186/s13049-014-0069-8

**Published:** 2014-11-22

**Authors:** Skule A Bakke, Morten T Botker, Ingunn S Riddervold, Hans Kirkegaard, Erika F Christensen

**Affiliations:** Department of Anesthesiology, Hospital of Southern Jutland, Southern Jutland, Denmark; Prehospital Research Department, Prehospital Emergency Medical Services, Central Denmark Region, Denmark; Research Center for Emergency Medicine, Aarhus University Hospital, Aarhus, Denmark

**Keywords:** Prehospital, Continuous positive airway pressure, Noninvasive ventilation, Respiratory failure, Acute pulmonary edema, Chronic obstructive pulmonary disease, Mortality, Hospital length of stay, Intensive care unit length of stay, Intubation rate

## Abstract

Continuous positive airway pressure (CPAP) and noninvasive ventilation (NIV) are frequently used inhospital for treating respiratory failure, especially in treatment of acute cardiogenic pulmonary edema and exacerbation of chronic obstructive pulmonary disease. Early initiation of treatment is important for success and introduction already in the prehospital setting may be beneficial. Our goal was to assess the evidence for an effect of prehospital CPAP or NIV as a supplement to standard medical treatment alone on the following outcome measures; mortality, hospital length of stay, intensive care unit length of stay, and intubation rate. We undertook a systematic review based on a search in the three databases: PubMed, EMBASE, and Cochrane. We included 12 studies in our review, but only four of these were of acceptable size and quality to conclude on our endpoints of interest. All four studies examine prehospital CPAP. Of these, only one small, randomized controlled trial shows a reduced mortality rate and a reduced intubation rate with supplemental CPAP. The other three studies have neutral findings, but in two of these a trend toward lower intubation rate is found. The effect of supplemental NIV has only been evaluated in smaller studies with insufficient power to conclude on our endpoints. None of these studies have shown an effect on neither mortality nor intubation rate, but two small, randomized controlled trials show a reduction in intensive care unit length of stay and a trend toward lower intubation rate. The risk of both type two errors and publication bias is evident, and the findings are not consistent enough to make solid conclusion on supplemental prehospital NIV. Large, randomized controlled trials regarding the effect of NIV and CPAP as supplement to standard medical treatment alone, in the prehospital setting, are needed.

## Introduction

Dyspnea is a frequent symptom among patients in the prehospital setting [1]. Common causes of nontraumatic dyspnea are congestive heart failure, pneumonia, chronic obstructive pulmonary disease, and asthma [1]. The application of advanced airway management and alternative devices in the prehospital setting has recently been defined as one of the top priority research questions in physician-provided prehospital critical care [2].

Continuous positive airway pressure (CPAP) and noninvasive ventilation (NIV) are often used in intensive care units for treating respiratory failure caused by acute cardiogenic pulmonary edema (ACPE) and acute exacerbation of chronic obstructive pulmonary disease (COPD). CPAP-systems apply positive airway pressure with only minimal differences in the pressure applied during inspiration and expiration [3]. The term NIV covers different forms of noninvasive positive pressure ventilation, which in contrast to CPAP can also ad extra inspiratory support driven by a ventilator, thereby giving positive pressure ventilation [3,4]. Standard medical treatment given for acute respiratory failure is diverse, depending on assumed cause and type of emergency medical staffing. It ranges from simple supplemental oxygen therapy to nitrates, diuretics, opioids, inhaled bronchodilators, and inotropic infusions. The worst cases can result in endotracheal intubation.

Recent Cochrane reviews show lower mortality and reduced intubation rate with the use of inhospital supplemental CPAP and NIV, compared to standard medical treatment alone, in patients with ACPE and exacerbations of COPD [5,6]. Lower intubation rates decrease the risk of complications related to endotracheal intubation and invasive ventilation, especially pulmonary infections [7-9].

Prehospital intubation is associated with high success-rates in physician-staffed services [10]. However, aspiration of gastric contents during intubation is reported more frequent in the prehospital setting than in the emergency department [11]. One study reports complications in 14% of prehospital advanced airway managements [12]. Especially vomiting, hypotension, and hypoxia do occur, but only a minor proportion of the patients in this study would have been suitable for CPAP/NIV as only 21% were intubated because of hypoxia. More than half of the patients had cardiac arrest. A prerequisite for successful noninvasive treatment is early initiation of CPAP or NIV [13,14]. Thus it is reasonable to believe that many patients would benefit from earlier initiation of noninvasive treatment, in the prehospital setting, to avoid intubation and improve patient outcome.

The objective of this systematic review of controlled studies was to examine, whether CPAP or NIV initiated in the prehospital setting reduce mortality, abbreviate hospital length of stay (H-LOS), abbreviate intensive care unit length of stay (ICU-LOS), or lower intubation rate when used as a supplement to standard medical treatment alone.

## Review

### Methods

Published studies relevant for this review were identified by a search in the databases PubMed, EMBASE, and Cochrane on April 4^th^ 2013 and updated January 19^th^ 2014. Our inclusion criteria were: Controlled studies examining the effect of supplemental prehospital CPAP or NIV, compared to standard medical treatment alone, in adult patients with acute respiratory failure of any cause. In PubMed the following search string was used: (“Continuous Positive Airway Pressure” [Mesh] OR “Noninvasive Ventilation” [Mesh] OR non invasive ventilation) AND (“Respiratory Insufficiency” [Mesh] OR “Pulmonary disease, Chronic obstructive” [Mesh] OR “Heart Failure”[Mesh] OR “Pulmonary Edema” [Mesh] OR “Asthma” [Mesh]) AND (“Emergency medical services” [Mesh] OR prehospital OR pre-hospital OR out of hospital).

We systematically excluded studies that did not meet the inclusion criteria in a hierarchical manner according to the following exclusion criteria:

1. Studies not regarding CPAP or NIV
2. Not prehospital setting
3. Not acute respiratory failure of any cause
4. Not a clinical trial
5. Not a controlled design comparing supplemental CPAP or NIV to standard medical treatment alone
6. Not adult patients (≥18 years)
7. Abstract only

First the title of a study, as it appeared from the search pages in the respective databases, was read and searched for the exclusion criteria described above. If a study could not be excluded based on its title, the abstract was read. Based on the abstract, we excluded studies that did not meet the inclusion criteria in the same hierarchical manner. If exclusion could not be done based on the abstract, the entire article was read. By this selection process, studies with inhome use of noninvasive ventilation for chronic pulmonary disorders, CPAP or NIV during intrahospital transport, expert opinions, editorials, reports, and case series were excluded. Duplications and conference abstracts were removed. Two reviewers independently carried out the searches, and discrepancies regarding exclusion were solved by consensus. Subsequently, a hand-search through references in the included studies, relevant reviews, and the “related citations” feature on PubMed was performed. Two reviewers independently extracted study details from the included articles, searching for our endpoints of interest: mortality, H-LOS, ICU-LOS, and intubation rate. Discrepancies regarding data extraction were solved by consensus. The included studies were independently evaluated by two reviewers according to the Scottish Intercollegiate Guidelines Network 50 (SIGN 50) checklist for randomized and/or controlled trials [15]. SIGN implements the Grading of Recommendations Assessment, Development, and Evaluation (GRADE) Working Groups approach within its methodology. The SIGN 50 checklists section one shows quality of evidence rated in one of four categories (ranging from “well covered” to “not addressed”). Section two starts by rating the methodological quality of the study, based on answers in section one and using the following coding system: High quality (++): The majority of criteria are met. There is little risk of bias. Results are unlikely to be changed by further research. Acceptable quality (+): Most of the criteria are met. There could be some flaws in the study with an associated risk of bias. Conclusions may be changed by further research. Low quality (−): Either most criteria are not met, or there are significant flaws relating to key aspects of study design. We were aware that included studies would be heterogeneous, but used the same checklist to assess all types of controlled studies in order to improve the systematic approach and critical evaluation.

To minimize bias, two reviewers evaluated each study independently. Differences in assessment were discussed and discrepancies solved by consensus with a third reviewer. We have presented the results from all studies, but our conclusion is based only on studies rated + or ++.

## Results

**Figure 1:**
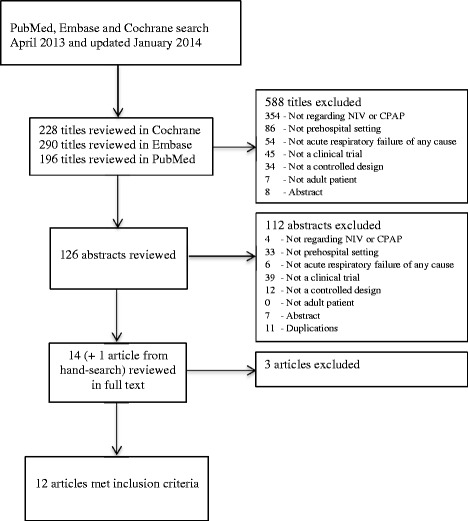
**Search flow diagram.**

We located 196 published studies searching PubMed, 290 studies searching EMBASE, and 228 studies searching Cochrane, yielding a total of 714 studies. Of these, 700 studies were excluded based on titles or abstracts. One study was found by hand-search [16] and, a total of 15 full-text articles, were read (Figure 1). We subsequently excluded one study that examines CPAP alone compared to CPAP and standard medical treatment [17]. Another study examines CPAP and medical treatment given at different time intervals, and not supplemental CPAP compared to standard medical treatment alone. It was therefore excluded [18]. Finally, one study was excluded because it is a cost-benefit analysis and not a clinical trial [19]. One study does not report if standard medical treatment was used in the intervention group and an email was sent to the corresponding author for clarification, but there was no reply. Based on our evaluation of the methods section, both intervention and control groups received medical treatment and the study was thus included for analysis [20]. Another study does not state a primary endpoint, but reports on endpoints of interest for our review and was therefore included [21]. Thus we included 12 studies for final analysis [16,20-30]. Of the included studies, eight studies examine CPAP as intervention [16,22-25,27,28,30] and four studies examine NIV as intervention [20,21,26,29].

Overview of study design and main findings of the studies can be found in Table 1. According to our evaluation of clarity and quality of the included studies, by use of the SIGN 50 checklist, eight studies either lack power to conclude on our outcomes of interest or entails high risk of bias [20-22,24-27,29]. Four studies have acceptable power and risk of bias [16,23,28,30]. The clarity and quality of the studies, according to the SIGN 50 checklist, is shown in Table 2.

**Table 1:** **Characteristics of included studies comparing standard medical treatment with supplementary CPAP or NIV**

**Study**	**Study Country**	**Intervention**	**Study design**	**Number of patients (N)**	**Crude mortality rates**	**Type of patients**	**Primary outcome**	**Primary result**	**Primary review outcomes**
							***secondary outcome(s)***	***secondary result(s)***	
Cheskes et al. [16]	Canada	CPAP	Observational, before-and-after	214 Interventions	17/214	ARF of any cause	Mortality, in‐hospital	7.9% vs. 7.5% (p=0.85)	Mortality: →
									H-LOS: N/R
				228 Controls	17/228				
									ICU-LOS: N/R
									IR: →
							*intubation rate*	*14.5% vs. 12.7% (p=0.59)*	
Dib et al. [27]	USA	CPAP	Retrospective, controlled	149 Interventions	N/R	Presumed ACPE	Prehospital treatment times	30 vs. 31 min (p > 0.01)	Mortality: N/R
									H-LOS: N/R
									ICU-LOS: N/R
									IR: ↓
				238 Controls	N/R		*clinical variables intubation rate*	*improvement (p’s <0.01) 2.6% vs. 4.6% (p<0.01)*	
Ducros et al. [30]	France	CPAP	Randomized, controlled multicentre	107 Interventions	8/107	Presumed ACPE	Combined criteria (successful)	Odds ratio 2.1 (1.2-4.0)	Mortality: →
									H-LOS: N/R
									ICU-LOS:→
									IR: →
				100 Controls	9/100		*Mortality, 48 hours mortality, in hospital Intensive care unit length-of-stay*	*odds ratio 1.4 (0.4-5.2) odds ratio 0.9 (0.4-2.5) 2 vs. 2 days (p=0.67)*	
Frontin et al. [28]	France	CPAP	Randomized, controlled	62 Interventions	6/60	Presumed ACPE	Treatment success	Odds ratio 1.19 (0.56-2.53)	Mortality: →
									H-LOS: →
									ICU-LOS: →
									IR: →
				62 Controls	7/62		*Intubation rate hospital length-of-stay Intensive care unit length-of-stay mortality, 30 days*	o*dds ratio 1,47 (0.23-9.23) 6 vs. 6 days (p=0,5) 8,2 vs. 8 hours (p=0,27) odds ratio 1.14 (0.36-3.65)*	
Gardtman et al. [22]	Sweden	CPAP	Observational, before-and-after	158 Interventions	18/158	Presumed ACPE	ACPE at admission	76% vs. 93% (p<0.0001)	Mortality: →
				158 Controls	18/158		m*ortality, 1 year*	22% vs. 27% (p=0.64)	H-LOS: →
									ICU-LOS: N/A
									IR: N/A
Garuti et al. [25]	Italy	CPAP	Prospective, observational with historical control group	35 Interventions	1/35	ARF of any cause	Mortality, adjusted	Odds ratio 0.06 (0.01-0.53)	Mortality: ↓
				125 Controls	30/125		*intubation rate hospital length-of-stay*	*no intubations 12 vs. 18.8 days (p<0.0001)*	H-LOS: ↓
									ICU-LOS: N/A
									IR: →
Hubble et al. [24]	USA	CPAP	Prospective, demographically controlled	120 Interventions 95 Controls	5.35%	Presumed ACPE	Intubation rate	Odds ratio 4.04 (1.64-9.95)	Mortality: ↓
					23.15%		*mortality, in hospital hospital length-of-stay*	o*dds ratio 7.48 (1.96-28.54) 5.58 vs. 7.66 days (p=0.755)*	H-LOS: →
									ICU-LOS: N/A
									IR: ↓
Thompson et al. [23]	Canada	CPAP	Randomized, controlled	35 Interventions	5/35	ARF of any cause	Intubation rate	Odds ratio 0.16 (0.04-0.7)	Mortality: ↓
				34 Controls	12/34		*mortality, in hospital Intensive care unit length-of-stay hospital length-of-stay*	*0.3 (0.09-0.99) 6.5 vs. 3 days 9 vs. 3 days*	H-LOS: →
									ICU-LOS: →
									IR: ↓
Craven et al. [29]	USA	NIV	Prospective, demographically controlled	37 Interventions	6/37	Presumed ACPE	Out of hospital treatment time	31.4 vs. 31.2 min (p=0.931)	Mortality:→
				25 Controls	2/24		*improvement in SpO2 hospital length-of-stay mortality, in-hospital*	*13.71% vs. 6.99% (p=0.037) 6.34 vs. 7.63 (p=0.48) 6/37 vs. 2/24 (p=0.462)*	H-LOS: →
									ICU-LOS: N/R
									IR: →
Roessler et al. [20]	Germany	NIV	Randomized, controlled	25 Interventions	1/24	ARF of any cause	Efficiency of treatment	100% vs. 80% (p=0.05)	Mortality: →
				26 Controls	2/25		*survival, 28 days intensive care unit length-of-stay hospital length-of-stay intubation rate*	96% *vs. 92% (p=1.0) 13.9 vs. 17.4 days (p=0,5) 1.3 vs. 3.7 days (p=0.03) 1 vs. 6 (p=0.66)*	H-LOS: →
									ICU-LOS: ↓
									IR: →
Schmidbauer et al. [21]	Germany	NIV	Randomized, controlled	18 Interventions	0/18	Presumed COPD	Dyspnea score	Improvement (p<0.001)	Mortality: -
				18 Controls	0/18		*respiration rate other clinical variables Intensive care unit length-of-stay*	*improvement (p=0.001) No diff in other clinical variables 59 vs. 185 (p=0.02)*	H-LOS: →
									ICU-LOS: ↓
									IR: →
Weitz et al. [26]	Germany	NIV	Randomized, controlled	10 Interventions	1/10	Presumed ACPE	SpO2 at hospital admission	97.3% vs. 89.5% (p=0.002)	Mortality: →
				13 Controls	1/13		c*linical variables Intensive care unit length-of-stay hospital length-of-stay*	*No diff clinical variables 1.7* ±*0.5 vs. 2.3* ±*0.6 days 8.2* ±*2.3 vs. 12.5* ±*1.8 days*	H-LOS: →
									ICU-LOS: →
									IR: N/A

All comparisons are intervention vs. control. Arrows showing; no difference →, improvement/reduction ↓.

(Continuous positive airway pressure, CPAP; Positive pressure ventilation, PPV; Acute respiratory failure, ARF; Acute cardiogenic pulmonary edema, ACPE; Chronic obstructive pulmonary disease, COPD; Intubation rate, IR; Mortality in-hospital, IHM; Oxygen saturation, SpO2; Respiration rate, RR; Clinical variables, CV; Not Available, N/A; Not Reported, N/R; Hospital Length Of Stay, H-LOS; Intensive Care Length Of Stay, ICU-LOS.

**Table 2:** **Clarity and quality of the included studies**

**Study**	**Cheskes et al.** **[** 16 **]**	**Dib et al.** **[** 27 **]**	**Ducros et al.** **[** 30 **]**	**Frontin et al.** **[** 28 **]**	**Gardtman et al.** **[** 22 **]**	**Garuti et al.** **[** 25 **]**	**Hubble et al.** **[** 24 **]**	**Thompson et al.** **[** 23 **]**	**Craven et al.** **[** 29 **]**	**Roessler et al.** **[** 20 **]**	**Schmiedbauer et al.** **[** 21 **]**	**Weitz et al.** **[** 26 **]**
1.1 The study addresses an appropriate and clearly focused question	••••	••	••••	••••	•••	••••	••••	••••	••••	••••	••	•••
1.2 The assignment of subjects to treatment groups randomised	NA	NA	•••	••••	NA	NA	NA	•••	NA	••••	••••	•••
1.3 An adequate concealment method is used	NA	NA	•	••••	NA	NA	NA	••••	NA	••••	••••	•
1.4 Subjects and investigators are kept ‘blind’ to treatment allocation	NA	NA	••	•••	NA	NA	NA	•••	NA	•	•	•
1.5 The treatment and control groups were similar at the start of the trial	•••	••	••••	••••	•••	••	••	•••	••	••••	•••	••
1.6 The only difference between the groups is the treatment under investigation	•••	••	••••	••••	••	••	••	•••	•••	••	••••	••
1.7 All relevant outcomes measured in a standard, valid and reliable way	••••	•••	•••	•••	••	•••	•••	••••	••••	•••	•••	•••
1.8 What percentage of the individuals or clusters recruited into each treatment arm of the study dropped out before the study was completed?	00	4/14911/238	11/10713/100	0/622/62	00	NRNR	10/12024/95	1/351/36	9/71 in total	2/51 in total	1/18 0/18	0/13 0/10
1.9 All the subjects are analysed in the groups to which they were (randomly) allocated	•••	NA	••••	••••	••••	NA	••••	••••	••	•••	•	•••
1.10 Where the study is carried out at more than one site, results are comparable for all sites	NA	••	•••	NA	NA	NA	••	NA	NA	NA	NA	NA
2.1 How well was the study done to minimise bias?	+	-	++	++	-	-	-	+	-	+	+	-
2.2 Taking into account clinical considerations, your evaluation of the methodolgy used, and the statistical power of the study, are you certain that the overall effect is due to the study intervention?	+	-	+	+	-	-	-	+	-	-	-	-
2.3 Are the results of this study directly applicable to the patient group targeted by this review?	+	-	+	+	-	-	-	+	-	-	-	-

Well covered ••••

Adequately addressed •••

Poorly addressed ••

Not addressed •

Not applicable NA

Not reported NR

Few/no criteria fulfilled -

Some criteria fulfilled +

All/most criteria fulfilled ++

### CPAP

In the eight studies comparing supplemental CPAP to standard medical treatment alone, the number of patients in the intervention groups ranges between 35 and 214 [16,22-25,27,28,30]. Three studies are randomized controlled trials [23,28,30]. One study is prospective but not randomized [24]. Three studies are before and after studies [16,22,25]. One study is a cross-sectional study, where outcomes of patients receiving CPAP are compared to outcomes from those not receiving this intervention [27]. In three studies comparing supplemental CPAP to standard medical treatment alone, a reduction in mortality is found [23-25]. No studies show a significant increase in mortality with administration of CPAP. One study shows a lower H-LOS [25] and another study reports no difference in H-LOS [22]. Three studies report no difference in ICU-LOS [23,28,30]. Intubation rate is reduced with supplemental CPAP treatment in three studies [23,24,27]. According to our evaluation of clarity and quality of the studies, four studies either lack power to conclude on our outcomes of interest [25] or entail high risk of bias [22,24,25,27] and were excluded from contribution to our conclusion. Four studies have acceptable power and risk of bias [16,23,28,30]. Of these, one smaller randomized controlled trial shows a reduction in both mortality and intubation rate [23]. The remaining three – one large descriptive study and two randomized and controlled studies show no effect on any of our outcomes of interest [16,28,30], but there is a trend toward lower intubation rate with supplementary CPAP in two of these studies [28,30]. The prehospital, inhospital, and overall intubation rates are shown in Table 3.

**Table 3:** **Intubation rates with supplemental prehospital CPAP/NIV compared to standard medical treatment alone**

**Study**	**Supplemental prehospital CPAP/NIV**	**Standard medical treatment alone**
**CPAP**		
**Cheskes et al. 2013**	**31/214**	**30/228**
**Prehospital**	**0/124**	**1/228**
**Inhospital**	**31/214**	**29/228**
**Ducros et al. 2011**	**3/107**	**6/100**
**Prehospital**	**NA**	**NA**
**Inhospital**	**NA**	**NA**
**Frontin et al. 2011**	**2/60**	**3/62**
**Prehospital**	**0/60**	**1/62**
**Inhospital**	**2/60**	**2/62**
**Thompson et al. 2008**	**7/35**	17/34*
**Prehospital**	**0/35**	**9/34**
**Inhospital**	**7/35**	**8/34**
Dib et al. 2012	NA	NA
Prehospital	4/149	11/238*
Inhospital	NA	NA
Gardtman et al. 2000	NA	NA
Prehospital	NA	NA
Inhospital	NA	NA
Garuti et al. 2010	0/35	14/125
Prehospital	0/35	14/125
Inhospital	0/35	0/125
Hubble et al 2006	10/120	24/95*
Prehospital	5/120	7/95
Inhospital	5/120	17/95
**NIV**		
Craven et al 2000	4/37	7/25
Prehospital	0/37	6/25
Inhospital	4/37	1/25
Roessler et al 2011	1/24	6/25
Prehospital	0/24	1/25
Inhospital	1/14	5/25
Schmidbauer et al 2011	3/18	7/18
Prehospital	1/18	0/18
Inhospital	2/18	0/18
Weitz et al 2007	NA	NA
Prehospital	NA	NA
Inhospital	NA	NA

*=statistical significant difference. Studies rated as having acceptable size and quality are bold.

### NIV

In the four studies comparing supplemental prehospital NIV to standard medical treatment alone, the number of patients in the intervention groups ranges between 10 and 37 [20,21,26,29]. Three of the studies have a randomized and controlled design [20,21,26]. The fourth study is a prospective controlled study with five rescue units administering bi-level positive airway pressure as intervention group and five units administering standard medical treatment alone as control group [29]. There was no difference in mortality with supplemental NIV compared to standard medical treatment alone. In two studies, a reduction in ICU-LOS is found [20,21]. One study reports no difference in ICU-LOS [26]. No difference in total H-LOS is found in any of the studies. No difference in intubation rate is shown in any of the studies. According to our evaluation of clarity and quality of evidence, all four studies lack power to make conclusions on our outcomes of interest [20,21,26,29] and two studies entail high risk of bias [26,29]. In two small studies with low risk of bias, a reduction in ICU-LOS and a trend toward decreased intubation rate with supplemental prehospital NIV is seen [20,21], see Table 3.

## Discussion

Our principal findings are: 1) One of four studies of acceptable quality shows a lower mortality and intubation rate with supplemental prehospital CPAP compared to standard medical treatment alone, and the remaining three are neutral. A trend toward lower intubation rate with supplemental prehospital CPAP is seen in two studies. 2) There is insufficient evidence to conclude on the use of supplemental prehospital NIV.

### CPAP

All the included studies are relatively small, and many lack the power to investigate hard endpoints like mortality. Three studies did indeed find a reduced mortality [23-25], but two studies had problems with the study design [24,25]. The risk of type two statistical errors in the included studies is high.

In the study by Cheskes et al. a trend towards increased mortality in the subgroups chronic heart failure, COPD and pulmonary edema was seen, but this was not found in the other studies and their result was not statistically significant [16].

Prehospital CPAP given as a supplement to standard medical treatment improves clinical endpoints like respiratory rate and arterial saturation [24,25,27], when compared to standard medical treatment alone. In studies where arterial gases were taken, an improvement was seen [25,30]. PaO_2_ improved and pH was higher in the intervention group in one study [25]. The other study showed lower PCO_2_ and normalization of pH, but did not report on PaO_2_ as an endpoint [30]. These findings, combined with the lower intubation rate [23] and trend toward lower intubation rates [28,30], indicate that CPAP may reduce prehospital intubation rates, but this needs to be verified. Whether or not this is beneficial, cannot be answered based on the current evidence.

Of the four studies with acceptable quality, two included patients with acute respiratory failure of any cause [16,23] and two included patients with ACPE [28,30]. The low number of studies does not allow us to conclude on differences between the conditions being treated for.

### NIV

There is insufficient evidence to conclude on the effect of supplemental prehospital NIV compared to standard medical treatment alone. The failure to demonstrate differences in mortality, intubation rate, and H-LOS could be caused by type two errors in these small studies. Thus this does not mean that the strategy should be abandoned in future research. Studies in patients with COPD indicate that CPAP decreases inspiratory work of breathing [31]. The addition of pressure support ventilation to positive end expiratory pressure, increases tidal volume in proportion to the amount of pressure applied and theoretically relives inspiratory muscles [32]. Thus theoretically, NIV should be advantageous and in all of the included studies measuring these, vital signs like respiratory rate improve when NIV is used. Arterial saturation significantly improves with supplemental NIV compared to standard medical treatment alone, including high fractions of inspired oxygen, in three of the studies included in this review [20,26,29]. In the fourth study, improvement in arterial saturation was more pronounced in the intervention group, but failed to reach level of significance [21]. We speculate that these improvements in vital signs may lead to a better patient outcome, but this was not demonstrated in the included studies – most likely because of low sample sizes. In both studies with acceptable risk of bias but low sample size, a trend toward lower intubation rate with supplementary NIV, compared to standard medical treatment alone, is found [20,21]. However, the small sample sizes in these studies prohibit us from making solid conclusions on the use of prehospital supplemental NIV.

### General considerations

The equipment used to administer CPAP or NIV includes an external pressure regulator (WhisperFlow [23,24,27,30]), a turbulent flow valve (Boussignac [28]), helmet CPAP (Castar-Starmed, Flow-meter [25]), a ventilatory system (Respironics 330000 [29]) and a portable ventilator (Oxylog 3000 [20,21,26]). The last two devices are used to administer NIV, and this equipment is technically more sophisticated than the equipment used to deliver CPAP. The medical staffing of the dispatched rescue teams in the included studies was heterogeneous. Physicians administered CPAP/NIV in five studies [20,21,26,28,30] and paramedics or emergency medical technicians administered CPAP/NIV in another five studies [16,23,24,27,29]. Nurses administered CPAP in one study [25] and in one study both paramedics and, for 25% of the time, nurses provided treatment with CPAP [22]. Further comparison and analysis of the equipment used, is beyond the scope of this review.

None of the studies included in this review report problems with safety, or with easy of use, when administrating CPAP or NIV, regardless of the treating clinicians’ qualifications. Only physicians provided NIV with the Oxylog 3000. The low number of included studies, and their varying study design, does not allow us to distinguish between conditions being treated for or to compare those who administered CPAP.

A recent systematic review and meta-analysis of randomized controlled trials on prehospital CPAP/NIV by Mal et al. finds a reduction in the need for inhospital invasive ventilation and mortality, with the use of prehospital noninvasive positive pressure ventilation [33]. The review by Mal et al. includes only randomized and controlled studies. In their review, seven heterogeneous studies on both CPAP and NIV, with a total of 632 patients, are combined in a meta-analysis. They also included one study that was excluded from our review [18]. This study by Plaisance et al. compares different treatment algorithms, both involving CPAP and medical treatment, at different time intervals. A large proportion of the studies included in the meta-analysis by Mal et al. were relatively small. This can increase the risk of overestimating the effect of the intervention due to publication bias, as small studies with negative findings are less likely to be accepted for publication. A recent review by Simpson et al. finds that prehospital CPAP/NIV appears to be safe and feasible therapy that results in faster improvement in physiological status, and that it may decrease the need for intubation, when compared to delayed administration in the emergency department [34]. They state there is weak evidence that NIV may decrease mortality, which is not in agreement with our findings. In their review, the majority of articles included are noncomparative descriptive studies only on ACPE, and they included three studies that were excluded from our review [17-19]. Simpson et al. recognized, but did not discriminate between different forms of NIV.

### Limitations

The risk of publication bias is, as for all reviews, a weakness of this study. No studies in non-English languages met our inclusion criteria, but among our excluded studies were studies published in German, Spanish, Japanese, French, and Russian. However, there is a risk that studies published in other languages than English to a lesser extend are indexed in the searched databases. This can theoretically produce an overestimation of the positive effects of CPAP or NIV [35].

We used the SIGN 50 checklist designed for randomized controlled trials, because this checklist is relevant when considering nonrandomized studies as well. These study evaluations inevitably involve a degree of subjective judgment.

The external validity of our review is difficult to outline as the included studies are from different parts of the world, with different geographic characteristics, different medical staffing, and with different structure of the emergency medical services. Standard medical treatment used in the included studies cannot be regarded as uniform, although medical treatment of acute exacerbations of COPD and ACPE is well established. This could make the results less comparable.

When considering all patients attended, few are intubated in the prehospital setting [12,36,37]. In the Scandinavian countries, as in the majority in Europe, physicians provide prehospital advanced airway management and have the ability to intubate the trachea on scene. The treating clinicians in the included studies are often paramedics and not experienced physicians – this may have influenced intubation rates [38].

The pressures applied when administrating CPAP or NIV varied among the included studies. Reported pressure settings when administrating CPAP in the included trials, ranged from 5 to 10 cm H_2_O. One study examining NIV adjusted pressures according to a predefined protocol, and up to 20 cm H_2_O support pressure was given [20]. Four studies did not report on pressure settings [16,21,22,29]. Different pressure levels could be clinically relevant when comparing interventions.

### Unanswered questions and future research

Supplemental prehospital CPAP seems to improve vital signs, compared to standard medical treatment alone, and there is a trend toward lower intubation rate. This needs to be confirmed in larger, randomized controlled trials, and whether it is beneficial in terms of lower mortality or morbidity also needs clarification.

Supplemental prehospital NIV, compared to standard medical treatment alone, also seems to improve vital signs, but it is unknown whether this affects patient outcome. With regards to what we know from the inhospital setting, it seems reasonable to include patients with acute exacerbations of COPD in future studies [6]. The application of NIV in the prehospital setting, and thereby early initiation, may be more advantageous in case of long distances to the receiving hospitals; this could also be a focus for future research. Interestingly, there were only few reported problems with mask-tolerance in studies included in this review – this is a well-described problem inhospital, and it is unlikely that these issues are smaller in the prehospital setting even if the treatment time is short. This subject – and the patients’ perception of mask treatment, in the prehospital setting, could also be a focus for future research [39,40].

## Conclusion

The current evidence shows no difference in mortality or hospital length of stay, but a trend toward reduced intubation rate with prehospital supplemental CPAP compared to standard medical treatment alone. This needs to be verified in larger, randomized controlled trials. The current evidence regarding prehospital supplemental NIV is scarce, and the conducted studies are too small to make reasonable conclusions, but justify further research.
